# A cross sectional study on nursing process implementation and associated factors among nurses working in selected hospitals of Central and Northwest zones, Tigray Region, Ethiopia

**DOI:** 10.1186/s12912-017-0248-9

**Published:** 2017-09-15

**Authors:** Zeray Baraki, Fiseha Girmay, Kalayou Kidanu, Hadgu Gerensea, Dejen Gezehgne, Hafte Teklay

**Affiliations:** 1 Department Nursing, Aksum University Health Science College, Aksum, Ethiopia; 2 Department Nursing, Mekelle University Health Science College, Mekelle, Ethiopia; 3 Department Public Health, Aksum University Health Science College, Aksum, Ethiopia; 4 Department Biomedical, Aksum University Health Science College, Aksum, Ethiopia

**Keywords:** Nurse, Nursing process, Implementation, Knowledge, Factor, Hospitals, Ethiopia

## Abstract

**Background:**

The nursing process is a systematic method of planning, delivering, and evaluating individualized care for clients in any state of health or illness. Many countries have adopted the nursing process as the standard of care to guide nursing practice; however, the problem is its implementation. If nurses fail to carry out the necessary nursing care through the nursing process; the effectiveness of patient progress may be compromised and can lead to preventable adverse events. This study was aimed to assess the implementation of nursing process and associated factors among nurses working in selected hospitals of central and northwest zones of Tigray, Ethiopia, 2015.

**Method:**

A cross sectional observational study design was utilized. Data was collected from 200 participants using structured self-administered questionnaire which was contextually adapted from standardized, reliable and validated measures. The data were entered using Epi Info version 7 and analyzed using SPSS version 20 software. Data were summarized and described using descriptive statistics and multivariate logistic regression was used to determine the relationship of independent and dependent variable. Then, finally, data were presented in tables, graphs, frequency percentage of different variables.

**Result:**

Seventy (35%) of participants have implemented nursing process. Different factors showed significant association. Nurses who worked in a stressful atmosphere of the workplace were 99% less likely to implement the nursing process than nurses who worked at a very good atmosphere. The nurses with an educational level of BSc. Degree were 6.972 times more likely to implement the nursing process than those who were diploma qualified. Nurses with no consistent material supply to use the nursing process were 95.1% less likely to implement the nursing process than nurses with consistent material supply.

**Conclusion:**

The majority of the participants were not implementing the nursing process properly. There are many factors that hinder them from applying the nursing process of which level of education, knowledge of nurses, skill of nurses, atmosphere of the work place, shortage of material supply to use the nursing process and high number of patient load were scientifically significant for the association test.

**Electronic supplementary material:**

The online version of this article (10.1186/s12912-017-0248-9) contains supplementary material, which is available to authorized users.

## Background

The nursing process is a systematic method of assessing, diagnosing, planning, delivering and evaluating individualized care for clients in any state of health or illness. Based on the scientific problem-solving method, it constitutes the foundation for nursing practice [1]. The nursing process has been described as a theory of how nurses organize the care of individuals, families and communities. Lydia Hall was the first person to introduce the concept of “nursing process” into nursing in 1955 while addressing a group of nurses in New Jersey. The theory of the nursing process has been largely accepted by nurses since 1967 [2–4].

The nursing process has become the basis of contemporary practice of a core component of nursing education as well as a point of reference in providing nursing care in many parts of the world. Arguably it is central to all nursing actions, applicable in any setting and within any frame of reference [5, 6]. In practice, however, not all steps are systematically implemented. Studies have revealed difficulties in establishing and using the nursing process within institutions during the last years especially in developing countries [7].

In Africa, many countries have adopted the nursing process, but problem are found in its implementation in the clinical setting. A study conducted in four African countries found that while nurses generally agree on the benefits of the nursing process, it is not commonly used in practice. The constraints identified by the study included, its time consuming nature, failure of nurse leaders to motivate others, shortage of staff and negative attitudes [8–10]. The government of Ethiopia places emphasis on quality of health service in general and quality of nursing care in particular [11]. However, there is still a gap in the implementation of nursing process among nurses working in hospitals. One recent study found that almost all of the nurses in Mekelle hospital reported that they did not use the nursing process during the provision of care to their patients [12]. Similarly a study conducted in Debremarkos and Finote Selam hospitals in Ethiopia showed that nursing process was implemented by only 37.1% [13].

There are different factors which affect the implementation of the nursing process in hospitalized setting. Institutional factors like organizational structure and facilities in both material and human resources are one of categories of factors that affect the implementation of the nursing process. The other category of factors is nursing factors for example knowledge, especially for formulation of nursing diagnosis in developing nursing care plans, skill, experience, interest and beliefs of nurses on the importance of the nursing process. These greatly affect the implementation [14–18]. Theoretically, if nurses fail to carry out necessary nursing care, then the effectiveness of patient progress may be compromised and can lead to preventable adverse patient events [19–21].

Although implementation of the nursing process was well investigated throughout much of the developed world, the issue has only rarely been researched in the developing countries including Ethiopia [9]. Therefore this study was intended to assess the implementation of nursing process and associated factors among nurses who work in hospitals in central and northwest zone, Tigray region of Ethiopia.

## Methods

### Study area and study period

The study was carried out in central and northwest Tigray region, Ethiopia from December to June, 2015. In this region there were eight hospitals; of which two were zonal, five were district level and one was a defense hospital. St. Mary and Sehul hospitals were zonal hospitals and each of them serve approximately about one million populations. On each of the two zonal hospitals there were more than 100 nurses on average. Adwa, Temben, Enticho, Shiraro and Mytsebri hospitals were a district hospital and each of them serve roughly 200,000 populations. In each of the five hospitals there were more than 60 nurses on average [22].

### Study design

A cross-sectional observational study design was conducted.

### Source of population

All staff nurses who have been working in St. Mary, Adwa, Suhul and Temben hospitals in central and northwest zones of Tigray region of Ethiopia.

### Study population

All sampled staff nurses who have been working in St. Mary, Adwa, Suhul and Temben hospitals in central and northwest zones of Tigray region of Ethiopia.

### Selection criteria

#### Inclusion criteria

Sampled nurses who were working in St. Mary, Adwa, Suhul and Temben hospitals which were found in central and northwest Tigray region of Ethiopia.

#### Exclusion criteria

Nurses who were temporary re-assigned, on annual leave, free service workers and those who declined to participate in the study.

### Sampling

#### Sample size determination

The sample size was determined based using the following assumption and a single population proportion (p) $\mathrm{n}=\frac{{\left({\mathrm{Z}}_{\infty /2}\right)}^2\mathrm{P}\ \left(\mathrm{P}\hbox{-} 1\right)}{{\mathrm{d}}^2}$was employed.

Where n is sample size desired, z^2^ is a standard normal score of 95% of confidence interval = 1.96, d is degree of accuracy or the margin of error = 0.05 and *p* = 37.1%, which was the population proportion of the nurses who implement the nursing process using the previous study in Debremarkos and Fnote Selam hospitals, Amahara, Ethiopia [13]. Since the total source of the population was less than ten thousand which was 368; then by using the correction formula and thus by adding 10% for the possible non-response rate, a total sample size of 200 was obtained. Proportional allocation to the size was employed to allocate the sample among the hospitals.

#### Sampling procedure

**Fig. 1:**
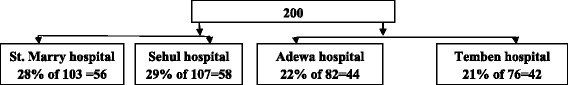
Schematic presentation of sampling procedure

Of the seven hospitals in the two zones, St. Mary, Adwa, Suhul and Temben hospitals were selected through a random lottery process. After proportion allocation of the sample among the selected hospitals was employed, the sampling frame of the hospitals was prepared. From the sampling frame, 200 nurses were selected by simple random sampling proportionally for each hospital. Since nurses have the right to decline and be considered as non-respondents, those nurses who declared their wish not to participate were treated as neutral (Fig. 1).

### Data collection procedure

Data were collected through a structured self-administered questionnaire and observational checklist. The data collection was collected by inviting 4th year Nursing Students of Axum University who were in internship practice in the hospitals under close supervision of the principal investigator from April 1 to May 2, 2015 to distribute and collect the surveys.

### Data collection tool

The questionnaires were adapted from a previous study. The validity of the data collection tool was determined by two experts and seniors with reliability of skill measurement questions, with a Cronbach’s alpha of 0.86. A structured English version questionnaire contains five main parts. Part I was used to collect data about nurses’ socio demographics, part II about nurse implementation status of the nursing process, part III about different factors, part IV about knowledge and part V about skill of nurses in nursing practice [15].

### Data quality assurance

To assure data quality, training and orientation was given for the data collectors by the principal investigator. The data collection tool was pre-tested on 5% of the participants two weeks before the actual data collection period in Mekelle hospital. Similarly necessary corrections and amendments were considered. During data collection, data collectors checked the data for its completeness and missing information at each point. Furthermore, data were checked during entry and compilation before analysis.

### Study variable

#### Dependent variable

- Implementation of nursing process

#### Independent variable

**Organization and facility related factors.**

Equipment access to nursing care.

Material supplies for nursing process.

Working environment.

Management system of the hospital.

Nurse’s patient load.

**Nurses related factor**

- Nurses demographics.
- Experience of nurses.
- Knowledge on implementation of nursing process.
- Skill of nurses on implementation of nursing process.
- Nurses dissatisfaction aspect of job
- Nurses strain during working time

### Operational definitions

**Nursing process implementation status**:- Nurses who answer “yes” for the six nursing process implementation questions and observed for their performance were as implementing the nursing process properly.

**Nursing practice skill**:- Those participants who scored >40 are highly skillful; 30–40 are moderately skillful, and <30 are low skillful groups out of 50 [15].

**Knowledgeable Nurses**:- Those participants who scored 80% were considered highly knowledgeable, 55–79.9% were considered moderately knowledgeable and **<**55% were low knowledgeable. For the purpose of the analysis which was based on literature, low and moderate knowledgeable and skilled nurses were combined together resulting in two categories only, high and low knowledgeable and skilled nurses [15].

### Data analysis procedure

After checking the data for its completeness, missing values and coding of questionnaires, data were entered into Epi info version 7, and analyzed using SPSS version 20. The statistical analysis was made at the 95% confidence level and with a 5% margin of error. The data were summarized and described using descriptive statistics. Multivariate logistic regression was used to determine the relationship between the independent and dependent variable. The independent variables; which were included in the multivariate logistic regression, were selected by doing a bivariate logistic regression with a cutoff point of *p*-value less than 0.3 and the goodness of fit model was checked by the Hosmer-Lemeshow statistic. Then these variables with *P*-value of <0.05 at 95% confidence interval (CI) were declared as statistically significant.

### Ethical considerations

Ethical approval was obtained from the research ethical review board of Mekelle University College of Health Sciences. An official letter of permission was obtained from Tigray Regional Health Bureau and was submitted to the respective selected hospitals. Written consent was obtained from each nurse prior to data collection. Participants were allowed to refuse or discontinue participation at any time up to data analysis. Information was recorded anonymously, and confidentiality and beneficence were assured throughout the study period (Additional file 1).

### Dissemination and utilization of result

The result of this research will be submitted to the Ministry of Health and Tigray Regional Health Bureau. The result will also be communicated with the hospitals where the research was done.

## Result

### Socio demographic characteristics of the respondents

In this study, 200 nurses were included. Among the participants 123 (61.5%) were female, the age of the participants ranged from 17 and 55 years with a mean age of 32.74 years and a standard deviation (SD) of ^±^8.94 years. Age distribution of study participants was not normally distributed, so median was used as the measure of central tendency. Approximately 53% of nurses were at age of 30 and below, or 106 nurses. And 111 (55.5%) held a Bachelor of Science in nursing degree at their level of education (Table 1).

**Table 1:** Sociodemographic characteristics of study participant (*n* = 200)

Characteristic	Frequency	Percentage
Sex		
➣ Female	123	61.5%
➣ Male	77	38.5%
Age		
➣ = <30	106	53%
➣ >30	94	47%
Ethnicity		
➣ Tigray	193	96.5%
➣ Other^a^	7	3.5%
Marital status		
➣ Single	77	38.5%
➣ Married	101	50.5%
➣ Other^b^	22	11%
Educational level		
➣ Diploma	89	44.5%
➣ Degree	111	55.5

^a^Amahara, Oromo & SPNN in Ethnicity

^b^Divorce, Widowed & Separated

### Nursing process implementation status

**Fig. 2:**
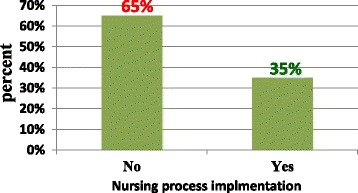
Nursing process implementation

Seventy (35%) of participants had the implemented nursing process in their practice while 130 (65%) had not implemented nursing process. Of these 124 (95.4%) nurses were not using the North America Nursing Diagnosis Association (NANDA). From those nurses who implemented the nursing process 28.6% were diploma educated while 71.4% were degree (or BSc) educated (Fig. 2).

### Organizational related factor

One hundred twenty (60%) of the nurses were taking care up to six patients per day with at mean of 6 patients per day (SD ^±^2 patients). 127 (63.5%) nurses were working overtime with a mean of 5.45 h (SD ^±^1.62 h). One hundred four (52%) of participants reported that they did not have necessarily equipments to perform a nursing care while 102 (51%) of participants reported that there was no consistent supply of materials to use the nursing process in their practice. Seventy five (37.5%) of nurses were working in a stressful atmosphere of the work place. One hundred fifty three (76.5%) of nurses responded there is nurse turnover in the hospital; of which 32.7% responded leaving to work for higher paying non-governmental organizations is the leading reason for the nurse turnover and 75.2% nurses reported that the nurse turnover had affected their nursing process implementation (Table 2).

**Table 2:** Organizational related factors (n = 200)

Characteristics	Frequency	Percentage
Nurses patient load per day		
➣ = <6	120	60%
➣ 7+	80	40%
Daily overtime working hrs		
➣ = <6	101	79.5%
➣ 7+	26	20.5%
All equipment to do nursing care		
➣ Yes	96	48%
➣ No	104	52%
Shortage of material supply for nursing process		
➣ Yes	102	51%
➣ No	98	49%
Have you work overtime		
➣ Yes	127	63.5%
➣ No	73	36.5%
Payment for the overtime		
➣ Yes	116	91.3%
➣ No	11	8.7%
The payment for overtime is enough		
➣ Yes	18	15%
➣ No	98	84%
Atmosphere of the work place		
➣ Stressful at a time	75	37.5%
➣ Disorganized	42	21%
➣ Well	31	15%
➣ Very well	52	26%
Nurses turnover in the hospital		
➣ Yes	153	76.5%
➣ No	47	23.5%
The reason of nurse turnover in the hospital		
➣ job&employee skill mismatch	31	20.3%
➣ Due to NGO’s attractive payment	50	32.7%
➣ Low access of short/long training	46	30.1%
➣ Less/no recognition for the work done	34	22.2%
➣ Bad management system of the hospital	25	16.3%
➣ Inferior facilities and tools	29	19%
Nurses that the nurse turnover affect the NPI		
➣ Yes	115	75.2%
➣ No	38	24.8%

### Nurses related factor

Work experience of participants varied from one to twenty nine years with a mean of 10 years and SD of 8 years. Forty eight (24%) of the nurses reported a great strain due to a symptomatic manager. One hundred fifty seven (78%) of the nurses were dissatisfied with their job; of which 66 (42%) were dissatisfied due to the patient workload and 131 (83.4%) nurses reported that dissatisfied had affected their use the nursing process. In regards to knowledge and skill of nurses to implement the nursing process; 79 (39.5%) of the nurses had low knowledge of the nursing process while 92 (46%) nurses were moderately skilled in the activity of nursing care (Table 3).

**Table 3:** Nurses related factors (n = 200)

Characteristics	Frequency	Percentage
Nurse strain during the working time		
➣ Rude(offensive) physicians	24	12%
➣ Harassing coworker	38	19%
➣ Unsympatic manager	48	24%
➣ When coworkers don’t do their task	34	17%
➣ The high patient load	34	17%
➣ No strain	22	11%
Nurses dissatisfaction with their job		
➣ Yes	157	78.5%
➣ No	43	21.5%
Reasons for nurse dissatisfaction on their job		
➣ Payment	61	38.9%
➣ Shortage of training	44	28%
➣ Management system of the hospital	45	28.7%
➣ Atmosphere of the work place	43	27.4%
➣ Communication with the ward staff	27	17.2%
➣ Having cared for so many patients	66	42%
Nurses that their dissatisfaction affect the NPI		
➣ Yes	131	83.4%
➣ No	26	16.6%
Nurses’ knowledge		
➣ Highly knowledgeable	46	23%
➣ Moderately knowledgeable	75	37.5%
➣ Low knowledge	79	39.5%
Nurses skill of nursing practice		
➣ Highly skilful	72	36%
➣ Moderately skilled	92	46%
➣ Low skill	36	18%

### Multivariate analysis result by factors affecting NP implementation

In bivariate analysis 12 variables met the criteria that *p*-value <0.3 in order to use multivariate analysis. Multicolinarity was near to one tolerance and <2 VIF. Six variables were significantly associated with *P*-value of <0.05 at 95% confidence interval. Nurses who have BSc. degree in their educational level are 6.972 times more likely to implement the nursing process than diploma-educated nurses when adjusting for all other factors (AOR = 6.972, 95% CI = 1.13–43.1). nurses with no consistent material supply to use the nursing process were 95.1% less likely to implement the nursing process than these nurses who had consistent material supply when adjusting for all other factors (AOR = 0.049, 95% CI = 0.008–0.286).

Nurses who worked in a stressful work place atmosphere were 99% less likely to implement the nursing process than nurses who worked in a very good atmosphere when adjusting for all other factors (AOR = 0.01, 95% CI = 0.001–0.091). Nurses who had a high patient load were 98.7% less likely to implement the nursing process than nurses who did not have a high patient load when adjusting for all other factors (AOR = 0.013, 95% CI = 0.001–0.13). Highly knowledgeable nurses were 15.09 times more likely to implement the nursing process than low knowledgeable nurses by adjusting all other factors (AOR = 15.09, 95% CI = 1.93–117.85). And highly skillful nurses were 22.16 times more likely to implement the nursing process than these low skilled nurses when adjusting for all other factors (AOR = 22.16, 95% CI = 3.01–122.64) (Table 4).

**Table 4:** Logistic regression analysis (n = 200)

Variable	Nursing process implementation		COR(CI 95%)	AOR(CI95%)
Sociodemographic	Yes	No		
Sex				
➣ Female	35	88	1	1
➣ Male	35	42	**2.095(1.155–3.801)**	2.272(0.566–9.127)
Age				
➣ = <30	40	66	1.293(0.720–2.321)	
➣ >30	30	64	1	
Marital status				
➣ Other	6	16	0.707(0.254–1.969)	
➣ Single	29	48	1.139(0.615–2.111)	
➣ Married	35	66	1	
Educational level				
➣ Diploma	20	69	1	1
➣ Degree	50	61	**2.828(1.517–5.270)**	6.972(1.128–43.093)*
Organizational related	Yes	No	COR(CI 95%)	AOR(CI95%)
Availability equipment to do nursing care				
➣ Yes	50	46	**4.565(2.429–8.580)**	2.066(0.445–9.797)
➣ No	20	84	1	1
Shortage of material supply for nursing process				
➣ Yes	14	88	**0.119(0.060–0.238)**	0.049(0.008–0.286)**
➣ No	56	42	1	1
Atmosphere of the work place				
➣ Stressful	5	70	**0.032(0.011–0.094)**	0.01(0.001–0.091)***
➣ Disorganized	9	33	**0.121(0.047–0.311)**	**0.021(0.002–0.180)** ^***^
➣ Well	20	11	0.808(0.315–2.074)	0.542(0.074–3.962)
➣ Very well	36	16	1	1
Dissatisfied due to MSH				
➣ Yes	9	36	**0.385(0.173–0.856)**	0.243(0.041–1.454)
➣ No	61	94	1	1
Nurses related factor	Yes	No	COR(CI 95%)	AOR(CI95%)
Nurses experience				
➣ = <7	40	63	1	1
➣ 8+	30	63	0.793(0.393–1.266)	0.755(0.153–3.731)
Nurses dissatisfied due to patient load				
➣ Yes	5	61	**0.087(0.033–0.230)**	0.013(0.001–0.13)***
➣ No	65	69	1	1
Nurses strain during the working time				
➣ Yes	56	122	**0.262(0.104–0.661)**	0.117(0.006–2.251)
➣ No	14	8	1	1
Nurses dissatisfied				
➣ Yes	40	117	**0.148(0.070–0.312)**	2.583(0.482–13.840)
➣ No	30	13	1	1
Nurses’ knowledge				
➣ Low knowledgeable	32	122	1	1
➣ Highly knowledgeable	38	8	**18.108(7.694–42.626)**	**15.085(1.931–117.85)** ^*^
Nurses skill				
➣ Low skill	21	107	1	1
➣ Highly skilled	49	23	**10.855(5.492–21.456)**	**22.16(4.01–122.64)** ^***^

* > 0.001, ** = 0.001, *** = 0.000

Bold Indicates: Significance Association

## Discussion

This study determines the level of nursing process implementation and associated factors among nurses in selected hospitals in the central and North West zone of Tigray, Ethiopia. In thise study of 200 nurses, there was a 35% of level of nursing process implementation.

This study had a lower level of nursing process implementation than a study conducted in Nigeria (in Abakaliki II and Calabar teaching hospitals) which showed a 67.2% and 62.7% respective use of the nursing process. In contrast, of this study the variation of the level of nursing process implementation may be because of the difference between the country level of development, sociodemographic factors for nurses and organizational structure and facilities. Also it is lower than study done in Addis Ababa, 2014 (52% of the level of implementation). Since Addis Ababa is the capital city of the country, the hospitals have better organizational facilities, human resources with the educational level of BSc and above, equipment access and material supply which contribute to better implementation of nursing process than the hospitals found in the regional towns. Therefore the above reasons may lead the discrepancy of the level of nursing process implementation [8, 10, 16].

The level of nursing process implementation from a study done on the assessment of the nursing process implementation and associated factors among nurses in Debremarkos and Fnoteselam hospitals, Amahara region in, 2014 was 37.1%. This is almost similar to the level of nursing process implementation of this study [13].

One hundred two (51%) respondents of this study said that irregular material supply to do nursing process affect the implementation of the nursing process. This result is supported by the study carried out in Abakaliki II teaching hospital Nigeria which is 32.7% of the respondents who said that the irregular supply of nursing process material affect nursing process implementation [8].

One hundred thirty one (83.4%) participants of this study reported their dissatisfaction due to excessive nurse patient ratio and other reasons had affected their use of nursing process. This result is in line with a cross sectional descriptive study in Teheran, Iran show that 84.1% of participants reported that dissatisfaction of nurses due to excessive nurse patient ratio and other reasons had affected the nursing process implementation. Again, this result is supported by the same study done in Addis Ababa that reported 54% of the participants caring for too many patients had affected the nursing process implementation [15, 16].

In this study from the total of 12 variables involved in the multivariate analysis; only six variables were significantly associated with *P*-value of <0.05 at 95% confidence interval. Not having consistent material supply to use the nursing process was significantly associated with the implementation of the nursing process. This result is also supported by a study done in Arbaminch Ethiopia, 2015 which reported that working in a hospital with low facility was negatively associated with the implementation of the nursing process [17].

This study also highly supported the study done in Addis Ababa and Arbaminch in selected hospitals, which found that nurses working in a stressful working environment were 64.3% and 77% less likely to implement the nursing process than those in an organized working environment, when adjusting for all other factors respectively. Similarly, in this study nurses working in a stressful atmosphere of the workplace were 99% less likely to implement the nursing process than nurses working at a positive work place environment when adjusting for all other factors [16, 17]. A stressful working environment decreases the impact of nursing care to the patient by degrading the implementation of the nursing process.

Highly knowledgeable nurses were 15.09 times more likely to implement the nursing process than low knowledgeable nurses when adjusting for all other factors. This results is similarly supported by the study done in Addis Ababa and Arbaminch, which reported that highly knowledgeable nurses were 38.91 and 8.78 times more likely to implement the nursing process than low knowledgeable nurses when adjusting for all other factors respectively [16, 17]. This shows that knowledge of nursing process enable for the nurses how they assess, diagnose, plan, implement and evaluate nursing practice.

## Conclusion

The majorities of the nurses were not implementing nursing process and had not used NANDA. Similarly they had low knowledge and were only moderately skilled in nursing process implementation. Level of education, knowledge of nurses, skill of nurses, atmosphere of the workplace, shortage of material supply to use the nursing process and high patient load had statistically significant association with the implementation of nursing process.

## Additional files

Additional file 1: Information sheet and questionnaire of the study. It is a data contained the information sheet for informed consent and a questionnaire of the study. (DOCX 25 kb)

Additional file 2: spss data of the study converted to excel**.** It is a data contained the spss filled data and convert to excel used for analysis of the data of the study. (XLSX 98 kb)

## Abbreviations

CI: Confidence interval
NANDA: North America nursing diagnosis association
SD: Standard deviation

## References

[1] Doenges ME, Moorhouse MF. Application of nursing process and nursing diagnosis: an interactive text for diagnostic reasoning. FA Davis; 2012.

[2] Adejumo P, Olaogun A (2009). Nursing Process: a tool for holistic approach to nursing care. West Afr J Nurs.

[3] Hermand T (2009). Nursing diagnoses: definitions & classification 2009–2011.

[4] Petro-Yura H. The nursing process: assessing, planning, implementing, evaluating. US: McGraw Hill: Appleton & Lange; 1988.

[5] Muszalik M, Kedziora-Kornatowska K (2005). Process of nursing as an active form nurse’s work with patient in therapeutic team–project of evidencing the process of looking after a sick person for students of nursing Faculty of Health Sciences, UMK Collegium Medicum in Bydgoszcz. Rocz Akad Med Bialymst.

[6] Scroggins LM (2008). The developmental processes for NANDA international Nursing diagnoses. Int J Nurs Terminol Classif.

[7] Pokorski S, Moraes MA, Chiarelli R, Costanzi AP, Rabelo ER (2009). Nursing process: from literature to practice. What are we actually doing?. Rev Lat Am Enfermagem.

[8] Edet A, Mgbekem M, Edet O. Professional Nurses’ Perception and Utilization of the nursing process at the University of Calabar Teaching Hospital (UCTH), Calabar, Nigeria. 2013.

[9] Sabona EA (2005). The perception on, and use of, the nursing process in four African Countries. Afr J Nurs Midwifery.

[10] Doubelegist: nursing process among nurses- factor affecting the implementation. 2013.

[11] FMOH, health Mo. addis ababa (2011). Nursing care practice standards, Reference manual for nurses and health care managers in Ethiopia.

[12] Hagos F, Alemseged F, Balcha F, Berhe S, Aregay A (2014). Application of nursing process and its affecting factors among nurses working in mekelle zone hospitals, Northern Ethiopia. Nursing Res Prac.

[13] Abebe N, Abera H, Ayana M (2014). The implementation of nursing process and associated factors among nurses working in Debremarkos and Finoteselam Hospitals, Northwest Ethiopia, 2013. J Nurs Care.

[14] Clarke SP, Aiken LH (2003). Failure to Rescue: needless deaths are prime examples of the need for more nurses at the bedside. Am J Nurs.

[15] Khorasgan I (2011). A survey on nursing process barriers from the nurses’ view of intensive care units. Iran J Crit Care Nurs.

[16] Mulugeta A (2011). Assessment on factors affecting implementation of nursing process among nurses in selected governmental hospitals, addis ababa, ethiopia. aau.

[17] Shewangizaw Z, Mersha A (2015). Determinants towards Implementation of Nursing Process. Am J Nurs.

[18] Momoh MA, Chukwu DO (2010). Factors that militante against the use of nursing. J Wilolud.

[19] De Vries EN, Ramrattan MA, Smorenburg SM, Gouma DJ, Boermeester MA (2008). The incidence and nature of in-hospital adverse events: a systematic review. Qual Saf Health care.

[20] Human TEI: Building a safer health system. Inst Med. 2000;112:5-8.

[21] Lucero RJ, Lake ET, Aiken LH (2010). Nursing care quality and adverse events in US hospitals. J Clin Nurs.

[22] FMOH, health Mo. addis ababa (2010). Guideline for implmemtation of patient reference system in Ethiopia.

