# Supplementary material for: A cross sectional study on nursing process implementation and associated factors among nurses working in selected hospitals of Central and Northwest zones, Tigray Region, Ethiopia
Source: BMC Nurs. 2017 Sep 15;16:54. doi: 10.1186/s12912-017-0248-9 (PMC5602869; doi:10.1186/s12912-017-0248-9)
Supplement: Supplementary file 1 — Information sheet and questionnaire of the study. It is a data contained the information sheet for informed consent and a questionnaire of the study. (DOCX 25 kb) [file 12912_2017_248_MOESM1_ESM.docx]

## Information sheet

Mekelle University, College of health sciences, department of nursing. Study on assessment of nursing process implementation and associated factors among nurses in hospitals of central and northwest zones of Tigray, Ethiopia, 2015

**Greeting:**

Hello, My name is_____________________. I am here today to collect data on nursing process implementation and associated factors among nurses. The study is being conducted by Mr. Zeray Baraki from Mekelle University, department of nursing. The objective of this study is to assess nursing process implementation and its associated factors here in central and northwest zones of Tigeray. I request you to take part in this study. Your cooperation and willingness are greatly helpful in the study. The study will be conducted through self administer questionnaires and you are being asked for a little of your time, about 25 min, to help us in this study. Your name will not be written in this form and will never be used in connection with any information. There is no possible risk associated with participating in this study. All information will be taken from you will be kept strictly confidential. Your participation is voluntary and you are not obligated to participate. If you feel discomfort with the data collection, it is your right to drop it any time you want. If you have questions regarding this study or would like to be informed of the results after its completion, please feel free to contact the principal investigator.

Address of the principal investigator:

Zeray Baraki

Cell phone: +251 921990232, E-mail: [zer2mun.dan@gmail.com](mailto:zer2mun.dan@gmail.com)

Are you willing to participate in this study?

1. Yes - ………………………….. Continue to the next page

2. No- …………………………… Skip to the next participant

## Questionnaire

**PART. I Socio Demographic Questions**

| **S/No** | **Items** | **Responses** | **Remark** |
| --- | --- | --- | --- |
| 1. | Sex | 1. Male 2. Female |  |
| 2. | How old are you? | Age________(in year) |  |
| 3. | What is your ethnicity? | 1. Tigraye 2. Amhara 3. SPNN 4. Oromo 5. Others _____________ |  |
| 4. | What is your current marital status? | 1. Single 2. Married 3. Widowed 4. Divorced 5. Separated |  |
| 5 | Educational level | 1. Diploma 2. Degree 3. Masters   4.Other ______________________ |  |

**PART – II Nursing process implementation Question**

| 6 | Does she/he use the nursing process in her/his daily nursing care? | 1. Yes 2. Not |
| --- | --- | --- |
| 7 | If the answer yes for QNo. 6 what types of assessment that he/she has used? | 1. Head to toes approach 2. Body system approach 3. Gordon’s functional health pattern approach |
| 8 | If the answer yes for QNo. 6, does he/she use NANDA for nursing diagnosis? | 1. Yes 2. No 3. If No what you use_____ |
| 9 | If the answer yes for QNo. 6, does he/she write a specific nursing care plan, including goal, expecting outcome and list of actions to be performed (list of nursing intervention)? | 1. Yes 2. No |
| 10 | If the answer yes for QNo. 6, does he/she perform and evaluate the nursing care plan? | 1. Yes 2. No |
| 11 | If the answer yes for QNo. 6, does he/she evaluate the implementation based on the expecting outcome? | 1. Yes 2. No |

**PART- III. Organizational and Nurses Related Questions:**

|  | **ITEM** | **Responses** |
| --- | --- | --- |
| 12 | How many years you did work in clinical area? | __________________________ |
| 13 | How many patients do you care per day? | ------------------------------------------------ |
| 14 | Do you have an interest to implement the nursing process in your daily nursing care? | 1. Yes 2. No |
| 15 | Do you believe in nursing process can qualify your nursing care? | 1. Yes 2. No |
| 16 | Do you have all equipment in your organization to do nursing care? | 1. Yes 2. No |
| 17 | Do you have a shortage of material supply to do nursing process? | 1. Yes 2. No |
| 18 | Have you worked over time? | 1. Yes 2. NO If ‘no’ skip Q.NO. 19 |
| 19 | If your answer ‘yes’, for QNo.18, how many hrs is it? | ____________ |
| 20 | If your answer ‘yes’, is that with payment? | 1. Yes 2. NO If ‘no’ skip Q.NO. 21 |
| 21 | If your answer ‘yes’, is the payment enough? | 1. Yes 2. NO |
| 22 | The greatest strain for you during your working time is/are? | 1. Rude(offensive) physicians  2. Harassing coworker  3. Unsympatic manager  4. When coworkers don’t do their task.  5. High patient load to care  6. No strain |
| 23 | How would you describe the atmosphere of your  Workplace? | 1. Stressful at times  2. Disorganized  3. Well  4. Very well 5. Others (specify)-------- |
| 24 | Is there any dissatisfying aspect of your job? | 1. Yes 2. No. If ‘no’ skip Q. 25 |
| 25 | If your answer is yes for QNo. 24, What is/are the dissatisfying aspects of your job? | __________________________________________________________ |
| 26 | If you are dissatisfied any reason from Q.NO.25, is it affect your nursing process implementation? | 1. Yes 2. NO |
| 27 | Is there staff turnover at the hospital | 1. Yes 2. NO |
| 28 | If the answer is yes fore QNo. 27, What do you think the causes of employee turnover? | 1. job&employee skill mismatch  2. Due to NGO’s attractive payment  3. Low access of short/long training  4. Less/no recognition for the work done  5. Others (specify)-------------------------- |
| 29 | If your answer is yes Q.NO.27, is it affect your nursing process implementation? | 1. Yes 2. No |
| 30 | If your answer is yes Q.NO.29, how it affect your nursing process implementation? | __________________________________________________________ |
| 31 | Is there patient turnover before completion of service? | 1. Yes 2. No |
| 32 | If the answer is yes for QNo. 31, What do you think the major reason of patient turnover? | 1. Miss-understanding of the modern medicines 2. Due to poor economic status 3. Due to long waiting time to get the service 4. Others (specify)----------------- |
| 33 | If your answer is yes Q.NO.31, is it affect your nursing process implementation? | 1. Yes 2. No |
| 34 | If your answer is yes Q.NO.33 how it influences your nursing process? | _____________________________________________________________ |

**PART- III. Knowledge Assessment**

| 35 | The first step in your nursing process is? | 1. Collecting subjective & objective data  2. Evaluating what has been done for the patient  3. Indicating the activities to be done  4. Directly intervening the problem |
| --- | --- | --- |
| 36 | The primary aim of Gordon approach is? | 1. Focuses on ethical principles  2. Focuses on patients' responses towards their illness  3. Focuses on the disease process/medical diagnosis  4. Focuses on patient’s attendant interest |
| 37 | Select from the given option that is not a component of the nursing process. | 1. Assessment  2. Implementation  3. Planning  4. Evaluation  5. Evidenced based practice  6. Nursing diagnosis |
| 38 | Your appropriate nursing diagnosis for a patient with Malnutrtion to prevent future complication is? | 1. Potential nursing diagnosis  2. Actual nursing diagnosis  3. Medical diagnosis  4. Laboratory investigation  5. Others (specify)-------------------------- |
| 39 | What makes the nursing diagnosis different from medical diagnosis? | 1. Nursing dx focuses on the diseases than others   Patient’s response   1. Nursing dx focuses on the patient’s response 2. Both focus on patient’s responses 3. Both have similar procedures 4. Others (specify)-------------------------- |
| 40 | Who is mandatory for the better accomplishment of nursing process? | 1. Physician 2. Patient’s family 3. Nurses  4. Patients 5. All are mandatory |
| 41 | What are your activities During your planning phase? | 1. Assigning priorities  2. Specifying expected outcomes  3. Recording the data of the patient  4. Specifying goals  5. Identifying interdependent interventions  6. All could be your activities except No. 3 |
| 42 | Select your role during the implementation phase of your nursing process? | 1.propose the interventions  2. Implementing the proposed interventions  3. Performing the planned interventions by excluding  Activity of daily living  4. Stop the phase if the initial implementations fail to Change patients’ problem. |
| 43 | Select that maybe not a guide for your nursing process evaluation? | 1. The nursing diagnosis  2. Collaborative problems  3. Priorities and nursing interventions  4. Expected outcomes 5. All could be guidelines |
| 44 | Fluid volume deficits related to unresolved vomiting & diarrhoea as evidenced by dry oral mucosa and sunken eyes. From the given nursing diagnosis, select the aetiology? | 1. Fluid volume deficit  2. Unresolved vomiting and diarrhoea  3. Dry oral mucosa and sunken eyes  4. Dehydration |

**PART IV: Skill Assessment**

Score the following activities according to the frequency of perform. Mark by using ‘x’.

**1. Not at all 2. Not really 3. Undecided 4. Somewhat 5. Very much**

|  | ACTIVITIES | 1 | 2 | 3 | 4 | 5 |
| --- | --- | --- | --- | --- | --- | --- |
| 45 | Ability to apply theories of nursing practice |  |  |  |  |  |
| 46 | Ability to maintain patient dignity, privacy and confidentiality(using nursing skills) |  |  |  |  |  |
| 47 | Ability to practice principles of health and safety, including morning and handling, and infection control; essential first aid and emergency procedures |  |  |  |  |  |
| 48 | Ability to safely administer medicine and other therapies; (using nursing skills, Interventions/ activities to provide optimum care) |  |  |  |  |  |
| 49 | Ability to consider emotional, physical, and personal care, including meeting the need for comfort, nutrition, personal hygiene and enabling the person to maintain the activities necessary for daily life; (using nursing skills, Interventions/ activities to provide optimum care) |  |  |  |  |  |
| 50 | Ability to perform a proper assessment by using one of the assessment methods. |  |  |  |  |  |
| 51 | Ability to identify the exact nursing diagnose from NANDA based on the assessment |  |  |  |  |  |
| 52 | Ability to develop nursing care plans, including goal, expecting outcome and a list of nursing intervention |  |  |  |  |  |
| 53 | Ability to perform the list of nursing intervention |  |  |  |  |  |
| 54 | Respond for the outcome by evaluating appropriate and individualized programs of care working in partnership with the patient, their caregivers, family and other health workers. |  |  |  |  |  |

##### ***THANKS AGAIN FOR YOUR PARTICIPATION!!!!!!!!***
